# Efficacy of three-dimensional fast spin echo T2-weighted magnetic resonance imaging (Cube) for evaluating the longitudinal spread of perihilar cholangiocarcinoma after endoscopic biliary stenting: a diagnostic study

**DOI:** 10.1097/JS9.0000000000003438

**Published:** 2025-09-24

**Authors:** Keita Sonoda, Yuta Abe, Yoichi Yokoyama, Junya Tsuzaki, Shigeo Okuda, Akihisa Ueno, Ryo Takemura, Minoru Kitago, Yasushi Hasegawa, Shutaro Hori, Masayuki Tanaka, Yutaka Nakano, Hajime Okita, Masahiro Jinzaki, Yuko Kitagawa

**Affiliations:** a Department of Surgery, Keio University School of Medicine, Shinjuku-ku, Tokyo, Japan; b Department of Radiology, Keio University School of Medicine, Shinjuku-ku, Tokyo, Japan; c Department of Diagnostic Radiology, NHO Tokyo Medical Center, Meguro-ku, Tokyo, Japan; d Division of Diagnostic Pathology, Keio University School of Medicine, Shinjuku-ku, Tokyo, Japan; e Clinical and Translational Research Center, Keio University Hospital, Shinjuku-ku, Tokyo, Japan

**Keywords:** endoscopic biliary stenting, multidetector computed tomography (MDCT), perihilar cholangiocarcinoma, three-dimensional fast spin echo T2-weighted magnetic resonance imaging (Cube)

## Abstract

**Background:**

Although accurate assessment of the longitudinal tumor spread is vital for the resection of perihilar cholangiocarcinoma (PHC), endoscopic biliary stent (EBS) placement can make imaging difficult. This study evaluated the diagnostic performance of three-dimensional fast spin-echo T2-weighted MRI (Cube) compared with multidetector computed tomography (MDCT) for determining PHC extension before and after EBS placement.

**Methods:**

This retrospective study included 91 patients who underwent surgical resection for PHC at a single center between 2016 and 2024 with available Cube and MDCT data. Four imaging scenarios were analyzed for each patient: MDCT without EBS, MDCT + EBS, Cube without EBS, and Cube + EBS. Radiological findings were compared with the pathological reference standard. Sensitivity, specificity, positive predictive value, negative predictive value, and accuracy were calculated for each scenario. Inter-reader agreement was determined using Cohen’s kappa coefficient.

**Results:**

MDCT without EBS showed 79.1% accuracy in defining tumor extension, which dropped significantly to 30.2% after EBS placement (*P* < 0.0001). Cube imaging showed 77.3% accuracy without EBS and 70.7% accuracy with EBS, significantly higher than MDCT with EBS (*P* < 0.0001). The evaluability rate of MDCT decreased from 98% to 37% after stenting, whereas Cube retained an 85% evaluability rate with EBS. The interreader agreement was moderate to substantial for MDCT without EBS (*κ* = 0.74) and Cube without EBS (*κ* = 0.72) but declined for MDCT with EBS (*κ* = 0.34).

**Conclusion:**

Cube may be the preferred choice to guide surgical planning for PHC in patients without high-quality MDCT images before stent placement. Collectively, these findings suggest that Cube could bring about a paradigm shift in imaging-based diagnosis of PHC.

**HIGHLIGHTS**

- We assessed longitudinal tumor spread in PHC resection using MDCT or Cube.
- EBS placement interferes substantially with MDCT.
- MDCT + EBS exhibits markedly lower accuracy than with Cube.
- Cube + EBS showed a minimal reduction in accuracy compared with Cube without EBS.
- Cube may be best for tumor assessment in patients with PHC with newly placed EBS.

## Introduction

Perihilar cholangiocarcinoma (PHC) is a challenging malignancy with a poor prognosis. Surgical resection yields a 5-year overall survival rate of approximately 27%, compared with 1.8 and 1.6% in patients receiving systemic therapy or best supportive care, respectively[1]. Achieving R0 resection is a key prognostic determinant^[2-4]^, emphasizing the need for accurate preoperative assessment of longitudinal tumor extension. Current international guidelines recommend multidetector computed tomography (MDCT) and magnetic resonance imaging (MRI) as first-line imaging modalities for PHC^[5–7]^ based on several retrospective reports^[8–12]^. However, accurately defining the tumor extent using MDCT or MRI in patients with endoscopic biliary stents (EBSs) can be difficult because of artifacts caused by the stent. Although biopsy may be used for diagnostic confirmation, robust evidence of its efficacy in this setting remains limited.

Three-dimensional fast spin-echo T2-weighted MRI (Cube) has demonstrated superior sensitivity and resolution in various clinical settings, including the evaluation of central nervous system lesions[13] and musculoskeletal diseases^[14,15]^. Despite these advantages, its utility for characterizing the longitudinal extent of cholangiocarcinoma has not been extensively explored. Therefore, this study investigated the diagnostic performance of Cube in assessing the longitudinal spread of PHC, with a particular focus on its utility in patients with EBS, primarily the plastic stents commonly used for preoperative drainage. By addressing limitations posed by stent-related artifacts, these findings may help refine preoperative planning and improve patient outcomes. This study is compliant with the TITAN Guidelines 2025[16].

## Material and methods

### Patients and tumor samples

This retrospective study reviewed the medical records of consecutive patients who underwent surgical resection for suspected PHC between January 2016 and May 2024. The exclusion criteria were as follows: (1) alternative final pathological diagnoses (including benign lesions, hepatocellular carcinoma, liver metastasis, neuroendocrine neoplasm, or leiomyosarcoma); (2) receipt of preoperative chemotherapy or chemoradiotherapy; (3) absence of Cube imaging; and (4) presence of a metallic stent.

Data on demographic and clinical characteristics, radiological and laboratory findings, operative variables, postoperative outcomes, and pathological results were collected from hospital records. The work has been reported in line with the STARD Checklist.

### Multidetector Computed Tomography

CT studies were performed using 64–320-slice MDCT systems (Discovery CT 750 HD, Revolution; GE Healthcare, Milwaukee, WI, USA; Aquilion ONE; Canon Medical Systems, Tochigi, Japan). The dynamic CT protocol was as follows: First, a noncontrast CT scan of the upper abdomen (including the liver and pancreas) was performed. The arterial, portal venous, and equilibrium phases were acquired using a bolus-tracking method after administering contrast medium at a dose of 600 mgI/kg for 30 s using a power injector (DualShot GX, Nemoto Kyorindo, Tokyo, Japan).

For the arterial phase, a region of interest was placed on the descending aorta, and imaging was initiated at the earliest possible time after 100 HU enhancement was detected. The portal venous phase was acquired 50–60 s after the arterial phase, followed by the equilibrium phase at 90 s.

The timing of MDCT acquisition was based on the clinical workflow. For patients presenting to our department prior to biliary drainage, MDCT was routinely performed before EBS placement. However, for patients referred from other institutions after stenting, a pre-EBS MDCT scan was often not available. Post-EBS MDCT scans were scheduled as part of the preoperative work-up, fitted in amongst other clinical examinations and treatments.

### Cube

MRI studies were performed using 1.5T (Optima MR450w, Singa Artist, Signa Voyager; GE Healthcare) and 3.0T (Singa Pioneer, Discovery MR750, Discovery MR750w, GE Healthcare) machines.

Cube was acquired using a navigator trigger. The imaging parameters were as follows: repetition time, dependent on respiration; echo time, maximum; echo train length 90–100; field of view, 300–380 mm; matrix; 224–320 × 288–416; number of excitations, 1; and acquisition time, 160 s (dependent on respiration).

Similar to MDCT, the timing of Cube imaging was dependent on clinical scheduling. For patients without EBS, Cube imaging was performed when feasible but was generally not considered as urgent as the initial MDCT. Post-EBS Cube scans were also scheduled whenever possible during the preoperative evaluation period.

### Definitions of the points of evaluation

Ten anatomical points (A–J) were used to determine tumor involvement (Fig. 2): (A) superior border of the pancreas, (B) confluence of cystic duct, (C) confluence of hepatic duct, (D) left hepatic duct, (E) right hepatic duct, (F) confluence of B2 + 3 and B4 (or B2 and B3 + 4), (G) B2 + 3 (or B2), (H) B4 (or B3 + 4), (I) anterior branch, and (J) posterior branch (Fig. 2). For subgroup analysis by bile duct location, points (A) and (B) were categorized as extrahepatic; points (C–F) as perihilar; and points (G–J) as intrahepatic.

### Radiological evaluation

Two experienced abdominal radiologists, who were blinded to the pathological findings and surgical details, independently evaluated each of the 10 points for tumor involvement using 4 imaging scenarios: MDCT without EBS, MDCT + EBS, Cube without EBS, and Cube + EBS. The readers evaluated each scenario in separate sessions spaced at least 1 month apart to minimize recall bias. The images were evaluated using a picture archiving and communication system viewer. MDCT used reconstructed axial images with slice thicknesses of 1 or 1.25 mm, and the extent of tumor spread was defined as regions showing wall thickening. Cube imaging evaluated axial images, with wall thickening with intermediate-to-low signal intensity considered indicative of tumor spread. Outcomes were classified as positive for cholangiocarcinoma, negative for cholangiocarcinoma, unevaluable (e.g., severe stent artifacts), or undefinable (owing to anatomical variations). Discordant results were resolved by consensus. Undefinable points were excluded from both inter-reader agreement calculations and comparisons with pathological findings. Unevaluable points were counted as misclassifications in the diagnostic performance analysis.

The evaluability rate was defined as follows:

(total points−unevaluable points−undefinable points)/(total points−undefinable points)

Inter-reader agreement was calculated using Cohen’s *κ* coefficient for each imaging scenario.

### Pathological evaluation

A single experienced pathologist (A.U.), blinded to the clinical and imaging data, assessed each of the same 10 points (A–J) in the resected specimens. The pathological results were classified as invasive cholangiocarcinoma, carcinoma *in situ*, negative for malignancy, unevaluable, or undefinable. If the hepatic margin was positive for cholangiocarcinoma, points in the remnant liver were considered unevaluable; otherwise, they were considered negative. Points classified as unevaluable or undefinable were excluded from the diagnostic performance analysis.

### Diagnostic performance analysis

Radiological findings (positive, negative, or unevaluable) from MDCT and Cube (±EBS) were compared with the pathological reference standard. Carcinoma *in situ* was considered negative. Sensitivity, specificity, positive predictive value (PPV), negative predictive value (NPV), and accuracy were calculated. Radiological and pathological findings of a representative patient are shown in Fig. 3 and Supplemental Digital Content (see Video, Supplementary Digital Content 1, available at: http://links.lww.com/JS9/F215, which demonstrates these findings).

**Figure 1.**
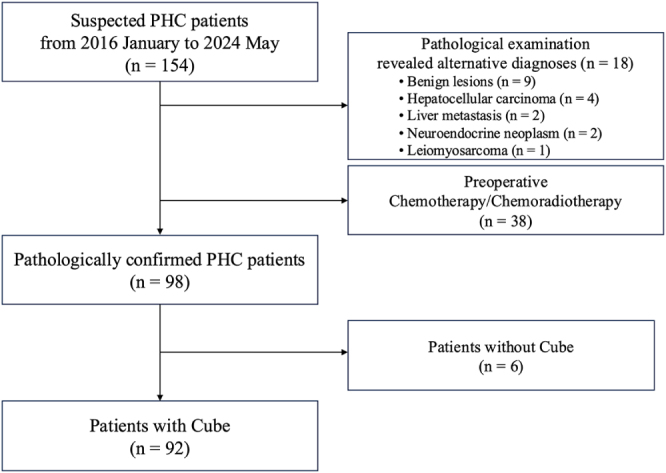
Flow chart of the study population. PHC, perihilar cholangiocarcinoma.

### Statistical analysis

All statistical analyses were performed using JMP version 18.0 (SAS Institute Japan, Tokyo, Japan). Categorical variables are presented as frequencies and percentages, whereas continuous variables are shown as medians with interquartile ranges. Cohen’s *κ* statistics were used to assess inter-reader agreement, and 95% confidence intervals (CIs) are reported. The diagnostic performance analysis calculated sensitivity, specificity, PPV, NPV, and accuracy. To test the hypothesis regarding the differences in accuracy among the four imaging groups, a χ^2^ test for pairwise comparisons was performed. Although *P* < 0.05 was initially set as the threshold for statistical significance, Bonferroni correction was applied because six pairwise comparisons were conducted; following this correction, the revised threshold was *P* < 0.0083. The subgroup analysis of bile duct location compared the accuracy across the extrahepatic, perihilar, and intrahepatic bile ducts using χ^2^ pairwise comparisons, again applying Bonferroni correction with *P* < 0.0083 as the threshold for significance. Finally, to assess the impact of the number of biliary stents, the accuracy of modalities with one stent was compared with that of modalities with two or three stents using a χ^2^ test. As these were two separates pairwise comparisons using one stent as the control group (1 vs. 2 stents, and 1 vs. 3 stents), a Bonferroni correction was not applied to prioritize avoiding a loss of statistical power. For this specific analysis, *P* < 0.05 was considered statistically significant.

## Results

### Patient selection

During the study period, 154 consecutive patients who underwent surgical resection for suspected PHC were identified. Of these, 18 patients were excluded due to alternative pathological diagnoses, and 38 patients were excluded for having received preoperative chemotherapy or chemoradiotherapy. This left 98 patients with pathologically confirmed PHC. From this group, six patients who did not undergo Cube imaging and one patient with a metallic stent were further excluded. Consequently, a final cohort of 91 patients was included in the analysis. This cohort consisted of 16 patients without EBS and 75 patients with a plastic stent. The patient selection process is summarized in Fig. 1.

**Figure 2.**
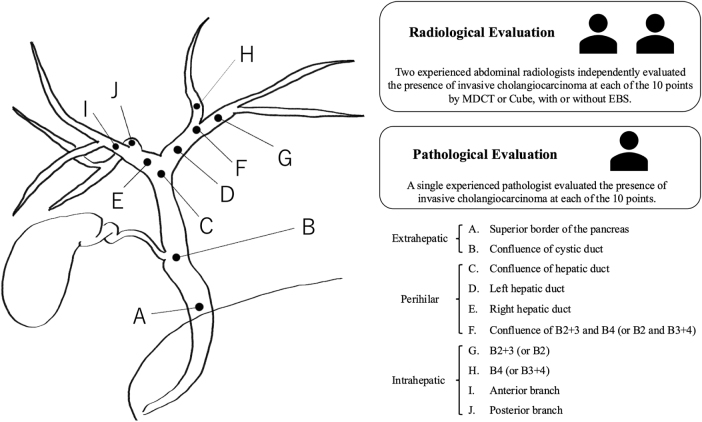
Scheme of the bile duct segments and distribution of the evaluation points. (A) superior border of the pancreas, (B) confluence of cystic duct, (C) confluence of hepatic duct, (D) left hepatic duct, (E) right hepatic duct, (F) confluence of B2 + 3 and B4 (or B2 and B3 + 4), (G) B2 + 3 (or B2), (H) B4 (or B3 + 4), (I) anterior branch, and (J) posterior branch. EBS, endoscopic biliary stent; PHC, perihilar cholangiocarcinoma.

**Figure 3.**
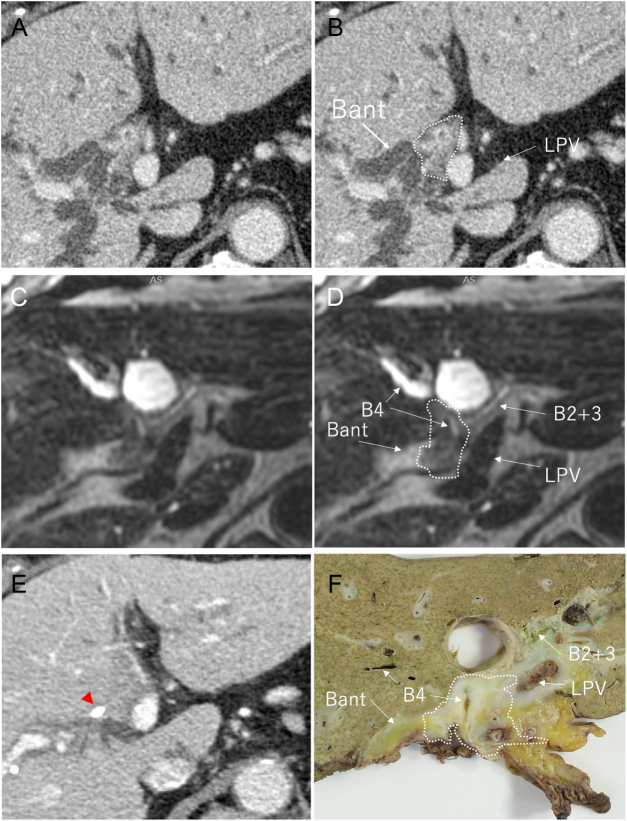
Radiological and pathological findings of a representative patient with Bismuth type IV PHC. Broken line, tumor; red triangle, EBS. (A, B) MDCT without EBS. (C, D) Cube + EBS. (E) MDCT + EBS, (F) Macroscopic findings. Broken line, tumor annotation based on the pathological finding. Bant, right anterior bile duct; B4, bile duct of segment 4; EBS, endoscopic biliary stent; LPV, left portal vein; MDCT, multidetector computed tomography; PHC, perihilar cholangiocarcinoma.

### Patient characteristics

Table 1 summarizes the clinical and pathological characteristics of the 92 included patients. The surgical procedures included right bisectionectomy (*n* = 43), right trisectionectomy (*n* = 2), left bisectionectomy (*n* = 22), left trisectionectomy (*n* = 13), and central bisectionectomy (*n* = 12). Of these, 14, 0, 5, 1, and 4 cases, respectively, were combined with pancreaticoduodenectomy. Portal vein and hepatic artery reconstruction were performed in 34 and 17 patients, respectively.

**Table 1:** Patient characteristics

Variables	Total (*n* = 91)
Sex (male)	64 (70.3)
Age (year)	73 [67–77]
Body mass index (kg/m^2^)	22.5 [20.0–24.0]
Days from imaging modality to surgery	
MDCT without EBS	49 [36–69]
MDCT with EBS	21 [10–51]
Cube without EBS	34 [22–49]
Cube with EBS	35 [25–47]
Operation time (min)	776 [686–941]
Blood loss (mL)	426 [251–653]
Type of resection (R2/R3/L2/L3/C2), [the number of combined PD]	42 [14]/2/22 [5]/13 [1]/12 [4]
Portal vein reconstruction	34 (37.4)
Hepatic artery reconstruction	17 (18.7)
Red blood cell transfusion	31 (34.1)
Bismuth type IV	27 (29.7)
Tumor differentiation (pap/well/mod/por)	5/16/57/13
T (1a/1b/2a/2b/3/4)	0/3/22/15/33/18
N factor (N0/N1/N2)	38/34/19
Stage (I/II/IIIA/IIIB/IIIC/IVA/IVB)	2/17/13/6/33/16/4
R (0/1/2)	67/22/2
HM (0/1/2)	81/8/2
DM (0/1/2)	89/2/0
EM (0/1/2)	75/15/1

C2, central bisectionectomy; EBS, endoscopic biliary stent; L2, left bisectionectomy: L3, left trisectionectomy; MDCT, multidetector computed tomography; PD, pancreaticoduodenectomy; R2, right bisectionectomy; R3, right trisectionectomy.

Continuous variables were expressed as medians and interquartile range.

### Radiological evaluation

The availability of each imaging modality was as follows: 71 MDCT scans without EBS, 65 MDCT scans + EBS, 28 Cube scans without EBS (3.0 T, *n* = 21; 1.5 T, *n* = 7), and 64 Cube scans + EBS (3.0 T, *n* = 47; 1.5 T, *n* = 17). The inter-reader agreement (Cohen’s *κ*) values were 0.74 for MDCT without EBS, 0.34 for MDCT + EBS, 0.72 for Cube without EBS, and 0.52 for Cube + EBS. The evaluation rates were 98% for MDCT without EBS, 37% for MDCT + EBS, 97% for Cube without EBS, and 85% for Cube + EBS (Table 2).

**Table 2:** Diagnostic results of radiological evaluation

	**MDCT without EBS**	**MDCT with EBS**	**Cube without EBS**	**Cube with EBS**
Total cases	71	65	28	64
Cohen’s *κ* (95% CI)	0.74 (0.69–0.79)	0.33 (0.27–0.38)	0.71 (0.63–0.79)	0.51 (0.45–0.57)
Evaluability rate	97.8% (673/688)	37.5% (238/635)	96.3% (262/272)	86.7% (539/622)

EBS, endoscopic biliary stent; MDCT, multidetector computed tomography.

### Diagnostic performance analysis

**Figure 4.**
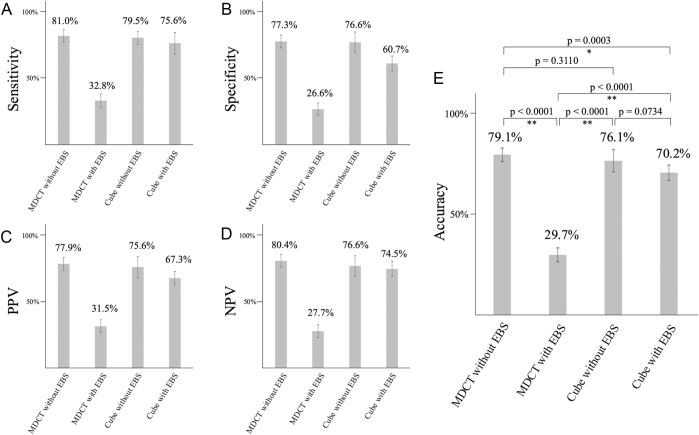
Diagnostic performance of each preoperative modality. (A) Sensitivity. (B) Specificity. (C) PPV. (D) NPV. (E) Accuracy. EBS, endoscopic biliary stent; MDCT, multidetector computed tomography; NPV, negative predictive value; PPV, positive predictive value. ^*^P < 0.083, ^**^P < 0.001.

Figure 4 shows the sensitivity, specificity, PPV, NPV, and accuracy of each modality. MDCT without EBS achieved an accuracy of 79.1% (95% CI, 75.9–82.1%), while MDCT + EBS demonstrated a significantly lower accuracy of 30.2% (95% CI, 26.7–33.9%). Cube without EBS showed an accuracy of 77.3% (95% CI, 71.8–82.0%). Cube + EBS had an accuracy of 70.7% (95% CI, 66.9–74.2%). Pairwise comparisons using Bonferroni correction showed significantly lower accuracy for MDCT + EBS compared with MDCT without EBS, Cube without EBS, and Cube + EBS (*P* < 0.0001). Cube with EBS also showed lower accuracy than MDCT without EBS (*P* = 0.0005).

### Comparison of accuracy according to bile duct location

**Figure 5.**
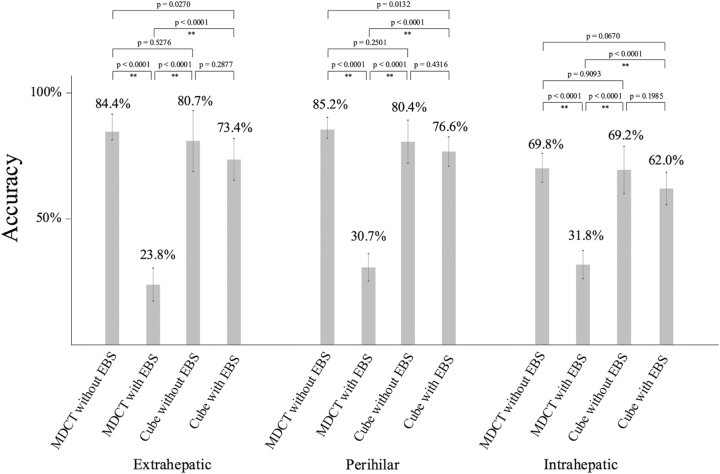
Subgroup analysis of accuracy according to bile duct location. EBS, endoscopic biliary stent; MDCT, multidetector computed tomography. **P < 0.001.

Figure 5 shows the results of the comparisons of accuracy across the extrahepatic, perihilar, and intrahepatic bile ducts. For extrahepatic bile ducts, the accuracy rates were 84.4% (95% CI, 77.5–89.5%) in MDCT without EBS, 23.8% (95% CI, 17.3–31.9%) in MDCT + EBS, 85.5% (95% CI, 73.8–92.4%) in Cube without EBS, and 72.7% (95% CI, 64.4–79.6%) in Cube + EBS.

For perihilar bile ducts, the accuracy rates were 85.2% (95% CI, 80.4–89.0%), 31.0% (95% CI, 25.6–37.1%), 79.6% (95% CI, 70.8–86.3%), and 77.9% (95% CI, 72.3–82.7%) for MDCT without EBS, MDCT + EBS, Cube without EBS, and Cube + EBS, respectively.

For intrahepatic bile ducts, the accuracy rates were 69.8% (95% CI, 63.9–75.2%), 32.7% (95% CI, 27.1–38.8%), 70.6% (95% CI,61.1–78.6%), and 62.1% (95% CI, 55.7–68.1%), respectively.

After Bonferroni correction (*P* < 0.0083), MDCT with EBS showed a significantly lower accuracy than the other modalities (*P* < 0.0001) for the extrahepatic, perihilar, and extrahepatic bile ducts. The other comparisons did not reach statistical significance.

### Comparison of accuracy according to the number of stents

Supplementary Digital Content Figure, Available at: http://links.lww.com/JS9/F216 shows the results of the comparisons of accuracy according to the number of stents. For MDCT with EBS, the accuracy rates were 32.3% (95% CI, 28.1–36.8%) for one EBS (*n* = 46), 25.7% (95% CI, 19.1–33.7%) for two EBS (*n* = 14), and 17.3% (95% CI, 9.1–30.7%) for three EBS (*n* = 5).

For Cube with EBS, the accuracy rates were 69.4% (95% CI, 64.9–73.5%) for one EBS (*n* = 46), 74.6% (95% CI, 66.6–81.2%) for two EBS (*n* = 14), and 63.9% (95% CI, 47.6–77.5%) for three EBS (*n* = 4).

MDCT with three EBS showed a significantly lower accuracy than that with one EBS (*P* = 0.038). The other comparisons did not reach statistical significance.

## Discussion

The results of this study demonstrated that the diagnostic accuracy of Cube for evaluating longitudinal tumor spread in PHC remains comparable to that of MDCT performed without EBS. Notably, Cube maintained its diagnostic performance even after EBS placement. To our knowledge, this is the first report to emphasize the efficacy of Cube in assessing PHC spread during EBS placement.

MDCT without EBS is widely considered the modality of choice for defining the longitudinal extent of PHC^[5–7]^. Senda *et al* demonstrated 80% accuracy in defining the longitudinal extension of PHC[17], a rate consistent with the findings of the current study. However, the diagnostic performance of EBS was significantly compromised. In urgent clinical scenarios such as cholangitis or obstructive jaundice caused by PHC, MDCT may be performed only after endoscopic intervention, leading to diagnostic challenges. Hosokawa *et al* reported that 41% of eligible patients underwent MDCT following biliary drainage, effectively comprising a “biliary drainage before referral” group[2]. In that cohort, although 69% of the patients underwent conventional CT scans before biliary drainage at the referring hospital, the image quality was insufficient to accurately assess PHC extension compared with high-quality MDCT scans. Consequently, to optimize the likelihood of R0 resection, patients with suspected PHC should ideally be referred to centers able to perform high-quality MDCT before biliary drainage.

In conventional magnetic resonance cholangiopancreatography (MRCP)-based evaluations of longitudinal PHC spread, diagnosis primarily relies on identifying upstream bile duct dilatation and downstream strictures[18]. However, evaluating a thickened ductal wall is challenging owing to heavy T2 contrast and fat suppression. Although Cho *et al* reported that MRCP performed after biliary drainage was comparable to MRCP performed before drainage[12], their patient cohort mainly comprised individuals with distal cholangiocarcinoma, with most patients undergoing pancreaticoduodenectomy, thus limiting the applicability of their findings to PHC in the presence of EBS. However, because Cube imaging does not incorporate fat suppression, it allows for the simultaneous evaluation of bile duct wall changes, which present as intermediate-to-low intensity in the lumen and periductal tissue. Furthermore, acquiring high-resolution three-dimensional images enables a more detailed assessment of longitudinal spread.

In the present study, Cube + EBS showed only a minimal reduction in accuracy compared with its use without EBS, whereas MDCT + EBS exhibited a markedly lower accuracy. Furthermore, the proportion of evaluable images was extremely high for MDCT and Cube without EBS, remained relatively high for Cube + EBS, and declined sharply for MDCT + EBS. The results of Cohen’s *κ* analysis were consistent with these findings, suggesting that EBS placement interferes substantially with MDCT than with Cube.

This difference in susceptibility to artifacts is further highlighted by our subgroup analysis based on the number of stents placed. For MDCT, diagnostic accuracy showed a decreasing trend as the number of stents increased, dropping significantly from 32.3% with one stent to 17.3% with three stents. In contrast, the accuracy of Cube remained high and stable, regardless of whether one, two, or three stents were in place. This result strongly supports our conclusion that Cube imaging is significantly more resilient to the cumulative artifact burden from multiple biliary stents compared to MDCT.

A practical consideration in clinical practice is that MDCT without EBS was performed in only 77% of the current cohort, highlighting the limited availability of MDCT before EBS placement. Consequently, Cube may be the best alternative for a significant proportion of patients who do not undergo MDCT before EBS placement.

Moreover, in the subgroup analysis stratified by bile duct location, only MDCT with EBS showed an inferior diagnostic performance for the extrahepatic, perihilar, and intrahepatic bile ducts; no significant differences were noted in the other comparisons. Therefore, the cube appears to be a useful modality for evaluating all bile duct segments, even after EBS placement.

In terms of technique, Cube is relatively straightforward to perform and does not require additional complex procedures beyond standard MRI protocols. Moreover, because it does not involve administering a contrast medium, Cube can be used in patients with chronic kidney disease or with allergies to iodine or gadolinium contrast. Thus, Cube is an attractive diagnostic modality, particularly for individuals who have not undergone MDCT before EBS placement.

Concurrent with advances in systemic therapies, including chemotherapy and immune checkpoint inhibitors, reports on preoperative chemotherapy or conversion surgery for cholangiocarcinoma have recently emerged. However, when evaluating the effects of such preoperative treatments, MDCT is limited to cases requiring EBS placement owing to artifacts caused by the stent. Consequently, the role of Cube in accurately assessing tumor response may soon become even more critical.

This study has several limitations. First, its retrospective design may have introduced selection bias. Second, the retrospective design led to unequal and variable patient numbers across the four imaging scenarios, which may have constrained the statistical power of our comparisons. Third, we excluded patients who received preoperative therapy to isolate the imaging characteristics without treatment-related confounders; however, this may limit the generalizability of our findings to the growing population of patients receiving neoadjuvant treatment. Fourth, the intervals between diagnostic procedures and surgery were not standardized, as detailed in Table 1. Furthermore, the time from stent placement to subsequent imaging was also variable. These temporal differences could have introduced bias due to tumor progression or fluctuating post-procedural inflammation. Fifth, although a 1-month washout period was implemented between reading sessions, the potential for recall bias cannot be fully eliminated, as radiologists may have remembered evaluating a prior scan for the same patient. Finally, this study focused solely on cholangiocarcinoma and regarded carcinoma *in situ* as negative for cholangiocarcinoma, as residual carcinoma *in situ* has not been reported to affect prognosis[19].

## Conclusion

Cube provides a diagnostic performance comparable to that of MDCT without EBS for evaluating the longitudinal spread of PHC and remains accurate even after EBS placement. Therefore, Cube may be the modality of choice to assess tumor extent in patients with newly placed EBS who have not undergone MDCT. Consequently, we propose Cube as a paradigm-shifting imaging technique for the diagnostic work-up of PHC especially for the patients after EBS placement.

## Data Availability

De-identified datasets generated and analyzed during the present study are available from the corresponding author (e-mail: abey3666@gmail.com) on reasonable request and with permission of the Keio University School of Medicine Ethics Committee.

## Research registration unique identifying number (UIN)

This research was registered to Japan Registry of Clinical Trials (JRCT). The trial ID is jRCT1030250176. https://jrct.mhlw.go.jp/en-latest-detail/jRCT1030250176

## Assistance with the study

We thank Editage (www.editage.com) for English language editing.
